# Do competing demands of physical illness in type 2 diabetes influence depression screening, documentation and management in primary care: a cross-sectional analytic study in Aboriginal and Torres Strait Islander primary health care settings

**DOI:** 10.1186/1752-4458-7-16

**Published:** 2013-06-06

**Authors:** Gill Schierhout, Tricia Nagel, Damin Si, Christine Connors, Alex Brown, Ross Bailie

**Affiliations:** 1 Menzies School of Health Research, Institute of Advanced Studies, Charles Darwin University, Darwin, Australia; 2 Northern Territory Department of Health, Darwin, Australia; 3 Centre for Chronic Disease, School of Medicine, University of Queensland, Brisbane, Australia; 4 Baker IDI, Central Australia, Alice Springs, Australia

**Keywords:** Diabetes mellitus, Type 2, Depression, Quality improvement, Indigenous health services

## Abstract

**Background:**

Relatively little is known about how depression amongst people with chronic illness is identified and managed in diverse primary health care settings. We evaluated the role of complex physical needs in influencing current practice of depression screening, documentation and antidepressant prescriptions during a 12-month period, among adults with Type 2 diabetes attending Aboriginal and Torres Strait Islander primary care health centres in Australia.

**Methods:**

We analysed clinical audit data from 44 health centres participating in a continuous quality improvement initiative, using previously reported standard sampling and data extraction protocols. Eligible patients were those with Type 2 diabetes with health centre attendance within the past 12 months. We compared current practice in depression screening, documentation and antidepressant prescription between patients with different disease severity and co-morbidity. We used random effects multiple logistic regression models to adjust for potential confounders and for clustering by health centre.

**Results:**

Among the 1174 patients with diabetes included, median time since diagnosis was 7 years, 19% of patients had a co-existing diagnosis of Ischaemic Heart Disease and 1/3 had renal disease. Some 70% of patients had HbAc1>7.0%; 65% had cholesterol >4.0 mmol1-1 and 64% had blood pressure>130/80 mmHg. Documentation of screening for depression and of diagnosed depression were low overall (5% and 6% respectively) and lower for patients with renal disease (Adjusted odds ratio [AOR] 0.21; 95% confidence interval [CI] 0.14 to 0.31 and AOR 0.34; 95% CI 0.15 to 0.75), and for those with poorly controlled disease (HbA1c>7.00 (AOR 0.40; 95% CI 0.23 to 0.68 and AOR 0.51; 95% CI 0.30 to 84)). Screening for depression was lower for those on pharmaceutical treatment for glycaemic control compared to those not on such treatment. Antidepressant prescription was not associated with level of diabetes control or disease severity.

**Conclusions:**

Background levels of depression screening and documentation were low overall and significantly lower for patients with greater disease severity. Strategies to improve depression care for vulnerable populations are urgently required. An important first step in the Australian Indigenous primary care context is to identify and address barriers to the use of current clinical guidelines for depression screening and care.

## Introduction

Clinically un-identified co-morbid depression amongst people with diabetes has been recognised as a major public health problem in a number of countries [1]. Screening for depression, and appropriate follow up and management are recommended in numerous diabetes and general practice guidelines worldwide, [2-5] and use of formal depression screening tools for diabetes has been incentivised for primary care in the UK Quality and Outcomes Framework (QOF) since 2006 [6]. Depression screening is also recommended in Australian General Practice, for those at higher than average risk, including Aboriginal and Torres Strait Islander middle-aged adults, and people with chronic illness [4,5,7]. Relatively little is known about the gap between evidence-based guidelines and routine practice in different primary care settings [8]. There is a particular dearth of information about adherence to guidelines in provision of health care to Aboriginal and Torres Strait Islander people, who have the poorest health of any group in Australia.

A number of depression and mental health risk screening tools have been developed for routine use and their suitability for use with Indigenous Australians has been assessed [9]. Psychometric properties of the PHQ-9, PHQ-2+ have been well studied in clinical populations generally; [10] amongst patients with diabetes; [11] and in Aboriginal and Torres Strait Islander populations [11-13]. The psychological distress domain (K-5) - a modified version of the Kessler Psychological Distress Scale-10 (K-10) has been used in survey settings in Australia amongst Indigenous adults [14]. Whilst the Kessler tools measure distress rather than depression, their scores correlate well with depression (and anxiety). A 13-item screening tool, including mental health risk, and alcohol and drug related risk has been developed and validated specifically for Indigenous populations [15]. Current practice in relation to the use of these and other tools amongst Aboriginal and Torres Strait Islander patients in general, and those with chronic illness, is unknown.

It has been shown that in primary care settings patients with more complex physical needs, such as multiple co-morbidities, are less likely to have major depression treated than those with more straightforward clinical presentations [16]. A few studies have reported on adherence to delivery of guideline-scheduled services for preventive and medical aspects of diabetes care in the context of other competing demands [17,18]. To our knowledge, no previous studies have explored the extent to which competing physical demands, including poorly controlled disease and disease severity, may be related to depression identification and care for people with diabetes specifically. This is an important issue because of the known associations between untreated depression and poor self management, poor cardio-metabolic control, and the development of diabetes-related complications [19-23] with potential to perpetuate a negative downward spiral. It is also a particularly important issue in respect of Aboriginal and Torres Strait Islander populations, who are known to have a disproportionate burden of both uncontrolled diabetes, and poor mental health, including depression. Our paper reports on a cross-sectional analytic study of clinical audit data from patients with Type 2 diabetes receiving routine care in Indigenous primary care centres in Australia. We report on evidence-practice gaps, and compare differences in depression screening, documentation and management between patients with different levels of disease severity, co-morbidity and diabetes control.

## Methods

### Study design and data source

We used clinical audit data from the Audit and Best-practice for Chronic Disease Extension (ABCDE) project, a national quality improvement initiative which aims to improve quality of care in a range of priority aspects of Indigenous primary health care in Australia, including chronic illness care, preventive care, and maternal and child health care [24].

While mainstream Australians access primary care through a universal system of general practice funded through Medicare, primary care systems for Indigenous people are more complex, with three major services sectors: Indigenous community controlled services, state and territory funded/operated services, and general practices. Indigenous community controlled services, and state and territory funded/operated services, and their service populations, are the setting for this project. These services (“health centres”) are at the forefront of providing primary health care, particularly in rural and remote settings where there are few medical practitioners. Health centres range in size from small centres (where in some cases the regular staff consists of only a single nurse, with other staff providing services through regular visits), through to large health centres staffed by a range of health professionals. What they have in common is the intention to increase access to comprehensive primary health care for Aboriginal and Torres Strait Islander populations. The areas served by health centres participating in this study were characterised by a high burden of chronic disease, low health literacy and high levels of socio-economic disadvantage.

Forty four health centres participating in the ABCDE project were included, located in 4 States/Territories. These were all the participating health centres that had conducted clinical audits of patients with diagnosed type 2 diabetes between April and December 2009, and therefore had all used an audit tool that included information on depression screening. The majority of the health centres (n=34/44) were located in rural or remote areas, 7 in regional areas and 3 health centres were located in urban areas. At each participating health centre, a random sample of 30 records of clients aged 16 years or older with a documented diagnosis of Type 2 diabetes was audited. An additional criterion for inclusion was that the client had to have lived in the community for a minimum of 6 of the previous 12 months. For centres with 30 or fewer clients with diabetes all records meeting the criteria were audited. Based on this sampling approach, we obtained a sample comprising 1174 adults (median age 51.5 years) with a clinical diagnosis of type 2 diabetes (median time since diagnosis 7 years). Some 36% of respondents were identified as having renal disease, 19% had a record of co-morbid ischaemic heart disease and 58% had a record of hypertension.

### Measures

Screened participants were those with a record of having been screened for depression using a formal named screening tool within the past 12 months. Standard screening tools included the K-5, K-6, K-10, PHQ-2+, PHQ-9 and Edinburgh Postnatal Depression Screening tool (EPDS). Screening status and documentation of depression, and other mental illness was extracted by trained data extractors through examining the patients’ medical summary sheets, hospital discharge summaries and other relevant summaries. Medication records were reviewed for evidence of a current prescription of antidepressant drugs.

For measures of cardio-metabolic control we extracted from clinical records the most recently documented values of Haemoglobin A1c (HbA1c), total cholesterol, blood pressure and albumin/creatinine ratio (ACR) within 12 months prior to the audit. We presented these indicators in a dichotomous format using cut-points for target levels for reference categories. Cut-points were selected based on diabetes clinical guidelines recommended for use in Australian Indigenous populations at the time of the study [25]. Documented diagnoses of renal disease, ischaemic heart disease, hypertension, and hyperlipaemia were recorded as present or absent, and treatment for diabetes was recorded as no medication (reference category), oral medications or insulin, with or without oral medications.

Other information extracted from clinical records included sex, age, smoking status and BMI. Information was also extracted on overall delivery of scheduled services through 13 service items which the clinical guidelines used across the states/territories recommend for people with diabetes. We constructed an overall measure of quality of clinical (medical) care through summing the 13 items and dividing the sum of services delivered by 13. The list of service items included in this measure, and its prior use has been previously reported [26].

### Statistical analysis

Means, proportions and medians were used to summarise data as appropriate. Our data had inherent multilevel, dependency structure, as data collected at the individual patient level were clustered within health centres, which in turn were clustered within jurisdictions. Multilevel random effects logistic regression techniques were used to explore various explanatory models of disease severity for depression screening, documentation and current prescription of antidepressants. Such models are suitable for this purpose as they allow (and account for) the possibility of residual correlation between individuals within groups. Having been screened for depression using a formal named tool; having a documented diagnosis of depression in the clinical record; and having a current prescription of antidepressant medication were treated as dependent variables in the models. Collinearity amongst the independent variables was explored prior to model fitting, removing variables where collinearity was indicated. All analyses were conducted using Stata software, version 10 (StataCorp, College Station, Tex, USA).

## Results

### Variation in screening, documentation and management of depression

Of the 44 health centres included in the study, 4 had used a named standard formal depression/distress screening tool in the preceding year. Amongst those health centres which had screened at least one client, there was wide variation in proportion of clients screened, with the lowest health centre having screened 37% of their clients and the highest 67%. Across all health centres, 5% of clients had been screened (Table 1).

**Table 1:** Variation in screening, documentation and management of depression by participating centres and jurisdictions

	**No. clients**	**No. health centres**	**% clients**	**Range between jurisdictions**	**Range between health centres**
*Total sample*	1174	44			
Screened for depression with named formal tool	55	4	5.0	0 to 52.3	0 to 66.6
Documented diagnosis of depression	70	24	6.0	2.5 to 24.8	0 to 33.3
Documented diagnosis of other mental illness	55	26	4.7	2.4 to 11.0	0 to 33.0
Current prescription of antidepressant drugs	65	28	5.5	2.8 to 19.3	0 to 33.0

The most common screening tool used was a module of 5 questions from the Kessler Psychological Distress Scale (“K-5”). K-5 scores ranged from 5 to 20 (mean=0.10.9, SD=2.99). Using the accepted cut off of 12, 18% (n=10) of those screened using this tool were classified as having high levels of distress, consistent with depressive or anxiety disorders. Further exploration of the data revealed that the majority of patients screened (69%) scored 11 or higher.

Regardless of whether or not they had been screened, 6% (70 people) of patients across 24 health centres had a documented diagnosis of depression, 5% had a diagnosis of another mental illness and 6% of patients had a current prescription of antidepressant medication. Documented depression ranged from 0 to 33% between health centres. Of the 65 people with a current prescription of antidepressant drugs, 39% (n=25) did not have a documented diagnosis of depression, nor documentation of any other mental illness.

### Demographic and clinical characteristics of screened, depression documented and medication-treated groups

From Table 2, depression screening and documentation were significantly higher for those overweight, compared to those not overweight. Overall, 49% of the sample with recorded BMI were in the overweight category (BMI>24) and 27% of the sample had a BMI of over 30 (obese). Participants aged 35–50 years were more likely to have been screened for depression than those 15–35 years. There was no significant difference in prescription of antidepressants or depression documentation by age group.

**Table 2:** Characteristics of the sample by depression screening, documentation and prescription of antidepressant medication

**Variables**	**Total sample**		**Screened with standard named tool**					**Documented depression**					**Prescription of antidepressant medication**				
	**n**	**%**	**n**	**%**	**OR**	**95% CI**		**n**	**%**	**OR**	**95% CI**		**n**	**%**	OR*	**95% CI**	
Total	1174	100	57	5.0				70	6.0				65	5.5			
Sex																	
Male	489	41.7	22	4.5	Reference			25	5.1	Reference			25	5.1	Reference		
Female	685	58.3	35	5.1	1.14	0.69	1.89	45	6.6	1.31	0.73	2.34	40	5.8	1.15	0.57	2.32
Age group																	
15-35 years	111	9.5	2	1.8	Reference			4	3.6	Reference			4	3.6	Reference		
35-49 years	434	37.0	20	4.6	2.63*	1.51	4.60	35	8.1	2.35	0.84	6.58	24	5.5	1.57	0.56	4.37
50-64 years	450	38.3	16	3.6	2.01	0.69	5.89	24	5.3	1.51	0.51	4.49	28	6.2	1.77	0.62	5.08
65+ years	179	15.2	19	10.6	6.47	1.26	33.27	7	3.9	1.09	0.35	3.40	9	5	1.42	0.45	4.44
Smoking status																	
Non-smoker	769	65.5	32	4.2	Reference			40	5.2	Reference			39	5.1	Reference		
Current smoker	405	34.5	25	6.2	1.52	0.62	3.72	30	7.4	1.48	0.89	2.46	26	6.4	1.28	0.83	1.99
BMI																	
BMI 24 or less	572	50.9	5	0.9	Reference			19	3.3	Reference			23	4	Reference		
BMI>24	551	49.1	52	9.4	11.82**	3.58	39.02	47	8.5	2.71*	1.35	5.46	39	7.1	1.82	0.87	3.80
Ischaemic heart disease																	
No	951	81.0	41	4.3	Reference			55	5.8	Reference			49	5.2	Reference		
Yes	223	19.0	16	7.2	1.72	0.71	4.14	15	6.7	1.17	0.7	2.10	16	7.2	1.42	0.72	2.82
Renal disease																	
No	742	63.2	48	6.5	Reference			58	7.8	Reference			47	6.3	Reference		
Yes	432	36.8	9	2.1	0.31**	0.17	0.56	12	2.8	0.34**	0.2	0.75	18	4.2	0.64	0.33	1.26
ACR level																	
<=3.4	270	33.8	28	10.4	Reference			17	6.3	Reference			17	6.3	Reference		
>3.4	528	66.2	11	2.8	0.18**	0.09	0.37	29	5.5	0.87	0.4	1.8	22	4.2	0.65	0.3	1.23
Hypertension																	
No	491	41.8	19	3.9	Reference			24	4.9	Reference			20	4.1	Reference		
Yes	683	58.2	38	5.6	1.46	0.75	2.87	46	6.7	1.41	0.8	2.34	45	6.6	1.66	0.96	2.88
Hyperlipidaemia†																	
No	629	53.6	19	3.0	Reference			29	4.6	Reference			96	26	Reference		
Yes	545	46.4	38	7.0	2.41*	1.30	4.46	41	7.5	1.68*	1.1	2.66	39	7.2	1.79*	1.07	2.97
Diabetes treatment																	
No medication	150	14.3	14	8.3	Reference			11	6.5	Reference			11	6.5	Reference		
Oral	678	63.8	29	3.9	0.44*	0.26	0.76	40	5.3	0.81	0.40	1.62	40	5.3	0.80	0.42	1.50
Insulin	222	21.9	14	5.4	0.63	0.38	1.04	19	7.4	1.14	0.50	2.58	19	7.4	1.26	0.53	3.01
HbA1c level																	
<=7.00%	301	30.0	28	9.3	Reference			27	9	Reference			19	6.3	Reference		
>7.00%*	702	70.0	25	3.6	0.36**	0.23	0.55	36	5.1	0.55*	0.34	0.88	35	5	0.78	0.46	1.31
Total cholesterol																	
<=4.00 mmol1-1	323	34.9	21	6.5	Reference			20	6.2	Reference			10	3.1	Reference		
>4.00 mmol 1-1	603	65.1	30	5	0.75	0.46	1.24	40	6.6	1.08	0.72	1.61	39	6.5	2.16	0.99	4.73
Blood pressure																	
<=130/80 mmHg	404	36.4	19	4.7	Reference			25	6.2	Reference			22	5.4	Reference		
>130/80 mmHg	706	63.6	37	5.2	1.12	0.63	1.98	41	5.8	0.93	0.66	1.33	37	5.2	0.96	0.54	1.70
*P<0.01; **P<0.001																	

Significance tests adjusted for clustering by health centre. Sample size might vary with missing data. BMI, body mass index.

† Further analysis (not shown in the table) revealed that the association between hyperlipidaemia and depression & screening was mediated by BMI; after adjusting for this interaction, no substantial effect of hyperlipidaemia on depression indicators remained.

Clients with a formal diagnosis of renal disease were less likely to have been screened for depression and less likely to have documented depression than those without renal disease. We also expanded the definition of renal disease/impairment to include patients with a recorded ACR>3.4. Using this expanded definition, those with renal disease/impairment (60% of patients with an ACR value documented) were also less likely to have been screened for depression than those without renal disease/impairment. People treated with oral hypoglycaemics had significantly lower levels of screening compared to those not on oral medications.

There was no relation between other co-morbid conditions and depression screening, documentation or current antidepressant prescription. Patients with a diagnosis of hyperlipidaemia were more likely to have been screened for depression, to have documented depression, and to have a current prescription of antidepressant medication than those without hyperlipidaemia. However this association was found to be mediated by BMI and did not retain significance in the multivariate model, which included adjustment for BMI, and other factors (Table 3).

**Table 3:** Adjusted effects of select disease severity indicators on depression screening, documentation and antidepressant prescription†

**Variables**	**Screened with formal tool**			**Documented Depression**			**Prescription of antidepressant drugs**		
	**AOR**	**95% CI**		**AOR**	**95% CI**		**AOR**	**95% CI**	
Renal disease									
No	Reference			Reference			Reference		
Yes	0.21**	0.14	0.31	0.34**	0.15	0.75	0.69	0.37	1.29
ACR level									
<=3.4	Reference			Reference			Reference		
>3.4	0.16**	0.08	0.32	0.77	0.38	1.51	0.50	0.23	1.03
Diabetes treatment									
No medication	Reference			Reference			Reference		
Oral	0.37**	0.23	0.61	0.80	0.38	1.69	0.99	0.49	2.02
Insulin/insulin+oral	0.48**	0.30	0.78	1.13	0.45	2.83	1.61	0.59	4.33
HbA1c level									
<=7.00%	Reference			Reference			Reference		
>7.00%*	0.40**	0.23	0.68	0.51**	0.30	0.84	0.80	0.48	1.33
Total cholesterol									
<=4.00 mmol1-1	Reference			Reference			Reference		
>4.00 mmol 1-1	0.83	0.48	1.44	1.00	0.67	1.51	2.05	0.89	4.72
Hyperlipidaemia†									
No	Reference			Reference			Reference		
Yes	1.86	0.85	4.09	1.23	0.47	3.22	1.28	0.61	2.69

†adjusted for sex, age, BMI and overall delivery of scheduled services; only those disease severity indicators significant in the bi-variate analysis are included

*P<0.01; **P<0.001.

### Clinical targets for diabetes management

Approximately 70% of the sample with complete clinical records (n=1003) had HbA1c higher than the recommended 7.0% level; 65% had cholesterol higher than the recommended 4.0 mmol1^-1^ level and 64% had blood pressure higher than the recommended 130/80 mmHg (Table 2). Those with HbA1c at higher than recommended levels were significantly less likely to have been screened for depression using a standard tool, and also significantly less likely to have documented depression than those with target HbA1c levels (Tables 2 and 3). We also checked for relationships between depression indicators and level of abnormal reading for each of the clinical indicators (not shown). Using a higher cut-off (>=5.5 mmolL^-1^) to define high total blood cholesterol, we found that those with high cholesterol were significantly less likely to have been screened for depression using formal tools (OR=0.39; 95% CI=0.21; 0.74) compared to those with target levels of cholesterol.

Table 3 shows that after adjustment for overall quality of care and relevant demographic factors, patients with renal disease, and those with higher than target levels of HbA1c remained significantly less likely to be screened for depression or have documented depression than patients without these conditions. Similarly, patients treated with oral medications and/or insulin were less likely to have been screened for depression than those not on pharmaceutical treatment for glycaemic control.

## Discussion

We found low overall use of formal depression screening tools, low levels of documentation of depression, and prescription of antidepressant medications.

After adjustment for potential confounders, patients with more severe diabetes-related disease were less than half as likely to have been screened for depression using a formal tool. Those with documented renal disease and those with HbA1c above target levels, were less than one third and less than one half as likely to have been screened for depression and to have documented depression respectively.

Our finding of lack of attention to depression overall, and a disproportionate lack amongst patients with competing demands for management of physical illness, is largely consistent with previous reports. Two US-based studies found that about 45%-51% of diabetes patients with depression were undiagnosed, [1,27] and that undiagnosed depression was more common amongst those with co-morbid cardiovascular disease (Prevalence Ratio [PR], 1.5; 95% CI: 1.2-1.9), and those in poor or fair health. (PR, 2.8; 95% CI: 2.1-3.6) [1]. Neither of these studies included an analysis of depression recognition gaps in relation to levels of cardio-metabolic control. One recent single-practice study that examined depression screening and treatment outcomes in patients with diabetes, found lower than anticipated use of formal tools, even within an incentivised system, and limited detection of new cases as a result of screening [28].

The lack of attention to depression overall in our study population, and lower levels for patients with worse physical health, may explain in part the poor achievement of clinical targets for diabetes management in our study population. The low rates of screening reported in our study raise questions about the utility and acceptability of available depression screening tools for Aboriginal and Torres Strait Islander patients. Amongst those screened, lower than expected case finding raises questions about what is currently being done about mental health care in routine primary health practice. It also raises questions about the circumstances under which routine screening for depression is likely to be most effective. Our finding that those overweight were more likely to be screened for depression than those not overweight, further suggests that clinicians may be informally triaging patients prior to screening. Various determinants of the utility of depression screening tools, including the intentions of providers in selecting whom to screen and why [29] need to be addressed in the context of providing comprehensive primary health care for Aboriginal and Torres Strait Islander people.

A previous study has demonstrated that the extent to which competing demands of physical illness influence attention paid to emotional and social well being is likely to be influenced by the type of depression treatment that is available, and whether or not this treatment is acceptable to patients [30]. These authors found that competing demands were a factor in determining care for depression, but only for patients for whom the depression care offered was acceptable. The implication is that improving depression care, including for Aboriginal and Torres Strait Islander patients, requires that models of care are culturally acceptable and flexible enough to respond to individual preferences, and that access to acceptable care is equitably available - including to those with complex physical needs. In our study, the finding that people with poorer cardio-metabolic control or disease severity were generally no more or less likely to have prescriptions of antidepressants, may similarly reflect some degree of non-acceptability of antidepressant treatment amongst some patients, or their providers. Racial disparities in antidepressant treatment of depressive symptoms in people with diabetes have been previously identified in the United States, [31] with these authors suggesting that such differences are likely to reflect differential treatment by health professionals, and/or cultural differences in acceptance of medical help for emotional distress. Some have suggested greater access to psychological interventions of proven efficacy is urgently needed. These may be of greater benefit than drug treatment, particularly for patients with chronic illness, where concerns over drug interactions can preclude use of antidepressant drugs. Higher levels of depression, distress and socio-political contributions to depression amongst Aboriginal and Torres Strait Islander peoples present additional considerations for the development of appropriate models of care in this setting [32]. There are few available guidelines to assist primary care providers in selecting the most appropriate interventions for this vulnerable population in general, or specifically for those with diabetes. Brief social interventions focusing on problem solving and awareness, such as those contained in the model developed through the Australian Integrated Mental Health initiative (AIMhi) in the Northern Territory, have shown promise in reducing distress measured with the K-10, and in improving other mental wellbeing outcomes using a number of different measures, [33] but such approaches have not been widely adopted.

The main limitations of our study are, first, health centres participated voluntarily in the study and were all enrolled in a continuous quality improvement intervention for chronic illness care. Therefore the data are not necessarily representative for the States/Territories involved, and may overestimate or underestimate attention to depression care. Also, the focus of our study is on depression care for people with Type 2 diabetes, and the findings may not be generalisable to patients with other chronic conditions. Second, we relied on clinical medical records to obtain data. Documented care may not be an accurate reflection of actual care. Third, our sampling approach, based on unweighted sex- and age-stratified samples, was designed to facilitate analysis of quality of care between communities, not to produce population estimates. Nonetheless our sampling approach is suitable for exploring the strength and direction of associations, reported as the main finding of this paper. Fourth, we did not have information on any non-drug treatment for depression that may have been made available to these patients. This may mean that we underestimated attention paid by primary care providers to emotional and social wellbeing. Fifth, the cross-sectional nature of our study limited our ability to disentangle potential relationships between depression screening, and having a diagnosis of depression. For example, if those with depression had greater disease severity, and were also less likely to have been screened, reported associations between disease severity and screening may have been due to confounding. On the other hand, if those with depression had greater disease severity, and were more likely to have been screened (consistent with a usual care pathway) the reported associations between depression screening and disease severity would be underestimated. Finally, owing to the nature of the data and the limited numbers of health centres using formal depression screening tools, we were unable to explore the impact of health centre and systems factors, which are likely to be additional important determinants of mental health care received in the primary care context.

## Conclusion

We found that patients with poorer cardio-metabolic control and greater disease severity were less likely to have attention paid to potential co-morbid depression. This finding may have relevance to other settings of high service load, and is a cause for concern given the increasing burden of non-communicable diseases worldwide, and the known disproportionate high risk of mental distress amongst people with chronic illness, including diabetes. Improving depression identification and care for vulnerable high risk populations is likely to require a range of strategies. These may include strategies to raise awareness of evidence-practice gaps in this area; to develop and implement optimal delivery systems for identification and management of depression; and to ensure that depression care is acceptable and flexible enough to accommodate individual and community preferences. An important first step in the Aboriginal and Torres Strait Islander primary care context will be to identify and address barriers to the use of current clinical guidelines regarding depression identification and management including for the most vulnerable populations with complex physical needs.

## Abbreviations

AIMhi: Australian integrated mental health initiative; QOF: Quality and outcomes framework; ABCDE: Audit and best practice for chronic disease extension; EPDS: Edinburgh postnatal depression screening; ACR: Albumin/Creatinine ratio; HbA1c: Haemoglobin A1c; K-5: Kessler psychological distress scale; BMI: (Body mass index).

## Competing interests

The authors have stated that there are no competing interests.

## Authors’ contribution

GS, RB and AB conceptualised the research question. RB, DS, CC and TN played a lead role in the development of clinical audit tools and protocols used in the study, and contributed to preparation of the manuscript. All authors read and approved the final manuscript.
